# Fitness components and natural selection: why are there different patterns on the emergence of drug resistance in *Plasmodium falciparum* and *Plasmodium vivax*?

**DOI:** 10.1186/1475-2875-12-15

**Published:** 2013-01-11

**Authors:** Kristan A Schneider, Ananias A Escalante

**Affiliations:** 1 Department of MNI, University of Applied Sciences Mittweida, Mittweida, Germany; 2 Department of Mathematics, University of Vienna, Vienna, Austria; 3 Center for Evolutionary Medicine and Informatics, Arizona State University, Tempe, USA; 4 School of Life Sciences, Arizona State University, Tempe, USA

**Keywords:** Fitness components, Natural Selection, Primaquine, Artemisinin based combination therapy, Malaria elimination, Gametocytogenesis

## Abstract

**Background:**

Considering the distinct biological characteristics of *Plasmodium* species is crucial for control and elimination efforts, in particular when facing the spread of drug resistance. Whereas the evolutionary fitness of all malarial species could be approximated by the probability of being taken by a mosquito and then infecting a new host, the actual steps in the malaria life cycle leading to a successful transmission event show differences among *Plasmodium* species. These “steps” are called fitness components. Differences in terms of fitness components may affect how selection imposed by interventions, e.g. drug treatments, differentially acts on each *Plasmodium* species. Thus, a successful malaria control or elimination programme should understand how differences in fitness components among different malaria species could affect adaptive evolution (e.g. the emergence of drug resistance). In this investigation, the interactions between some fitness components and natural selection are explored.

**Methods:**

A population-genetic model is formulated that qualitatively explains how different fitness components (in particular gametocytogenesis and longevity of gametocytes) affect selection acting on merozoites during the erythrocytic cycle. By comparing *Plasmodium falciparum* and *Plasmodium vivax,* the interplay of parasitaemia and gametocytaemia dynamics in determining fitness is modelled under circumstances that allow contrasting solely the differences between these two parasites in terms of their fitness components.

**Results:**

By simulating fitness components, it is shown that selection acting on merozoites (e.g., on drug resistant mutations or malaria antigens) is more efficient in *P. falciparum* than in *P. vivax*. These results could explain, at least in part, why resistance against drugs, such as chloroquine (CQ) is highly prevalent in *P. falciparum* worldwide, while CQ is still a successful treatment for *P. vivax* despite its massive use. Furthermore, these analyses are used to explore the importance of understanding the dynamic of gametocytaemia to ascertain the spreading of drug resistance.

**Conclusions:**

The strength of natural selection on mutations that express their advantage at the merozoite stage is different in *P. vivax* and *P. falciparum*. Species-specific differences in gametocytogenesis and longevity of gametocytes need to be accounted for when designing effective malaria control and elimination programmes. There is a need for reliable data on gametocytogenesis from field studies.

## Background

Malaria burden has made this disease a significant barrier for reaching global development. Malaria, however, is not one disease. Whereas *Plasmodium falciparum* is responsible for most cases in Africa, endemic regions outside of Africa are characterized by the presence of *Plasmodium vivax*, the second most important malaria parasite in terms of its morbidity. Although they are found in sympatry across endemic regions worldwide, there are well-known differences between *P. falciparum* and *P. vivax*. Indeed, they differ in key aspects of their basic biology, geographic range, and evolutionary origins [1-4]. Such distinctive characteristics affect control and elimination managements of these two malarias; specifically in terms of (i) treatment of their associated clinical diseases and (ii) the design of new control tools, such as choosing candidates for anti-malarial vaccines.

Regardless of these noticeable biological differences, it has not been properly discussed yet how interventions acting as selective forces (e.g. anti-malarial drugs) differentially affect each species. Such assessment is important considering that malaria elimination will face areas where these two parasites are prevalent. Even in Africa, where *P. vivax* is rightfully neglected when compared with the impact of *P. falciparum*, there is increasing evidence indicating that this parasite is present, but at a lower prevalence [5,6].

An example of differences between these two species, in terms of emerging adaptations hampering the efficacy of interventions, can be found in the onset of chloroquine (CQ) resistance. Whereas in *P. falciparum* CQ resistance originated and spread worldwide as result of several independent events [7], it remains the drug of choice for treating *P. vivax* in most areas worldwide*.* CQ-resistant *P. vivax* strains are typically present – if at all – at low prevalence, and resistance-levels are low compared with *P. falciparum*[8]. Why did drug resistance emerge slower in *P. vivax*? There are *ad hoc* explanations for this observation, e.g., resistance may have a greater cost in *P. vivax* or that CQ targets a different pathway in this parasite. Although these explanations are undoubtedly intuitive, they might confuse other mechanisms. Likewise, there is extensive evidence of balancing selection acting on major malaria antigens expressed in the merozoite stage in *P. falciparum,* whereas in *P. vivax* such patterns are harder to detect [9-12]. Again, it could be argued that the immune system does not necessarily recognize the same antigens equally efficiently in both parasites. Moreover, there are some species-specific proteins involved in the invasion of the red blood cells that could alter the overall immune response ([13] and references within there).

Regardless of the intellectual merit of these *ad hoc* explanations, it is worth exploring alternative arguments based on formal foundations; this allows proposing hypotheses susceptible to be tested with empirical data. Consequently, operational research will be enriched by providing new perspectives that will support sustainable evidence-based malaria-elimination programmes. Following this logic, this article discusses how the distinct biological characteristics of different *Plasmodium* species may explain, at least in part, the differences observed in terms of patterns associated with some forms of positive selection.

Successful transmission of malaria is characterized by many steps, which are referred to as fitness components. Although, malaria fitness in terms of successful transmission can be roughly summarized in the same way across all species (e.g. number of infected mosquitoes per sporozoite in the original infection), the relative weights of the involved fitness components differs among them due to species-specific characteristics. For example, *P. vivax* has hypnozoites and *P. falciparum* produces gametocytes relatively late and with a longer lifespan when compared with *P. vivax*[1,14-16]. Thus, interventions will likely affect the dynamic of mutations that could confer an advantage to the parasite (e.g. drug tolerance or resistance) differently in each species even if those mutations potentially offer the same relative metabolic advantage. Consequently, it could be hypothesized that differences in fitness components could make selection acting on the erythrocyte stages less efficient as an evolutionary force in one parasite species when compared with the other. This is a fundamental question in order to ascertain the effect of selection on the dynamic of mutations that could have a global health impact. Whereas there are several differences in term of fitness components between the two parasite species, by proposing a population-genetic model, this investigation specifically explores how differences in gametocytaemia in terms of their time of production and lifespan may affect the dynamics of an advantageous mutation expressed in the merozoite stage. Real examples of such mutations are the ones conferring drug resistance but similarities could be made in the case of variants associated with vaccine evasion or even polymorphisms maintained by balancing selection. However, for simplicity, this investigation focuses on mutations associated with resistance (or tolerance) to drugs acting at the merozoite stage. Overall, this investigation suggests that positive selection is by far less efficient as a driving force in *P. vivax* in mutations that express their metabolic (or immunologic) advantage during the merozoite stage. Although, this investigation concentrates only on the two clinically most relevant human malarias, the models and reasoning are valid for comparisons between any two human malaria species with obvious modifications. The results presented here are further discussed in terms of the importance of understanding gametocytaemia, not only in terms of its pivotal role in malaria transmission, but also in ascertaining the effect that natural selection has on the parasite population in terms of spreading mutations that may compromise the lifespan of tools used for control and elimination.

## Methods

### Basic terminology and assumptions

**Figure 1:**
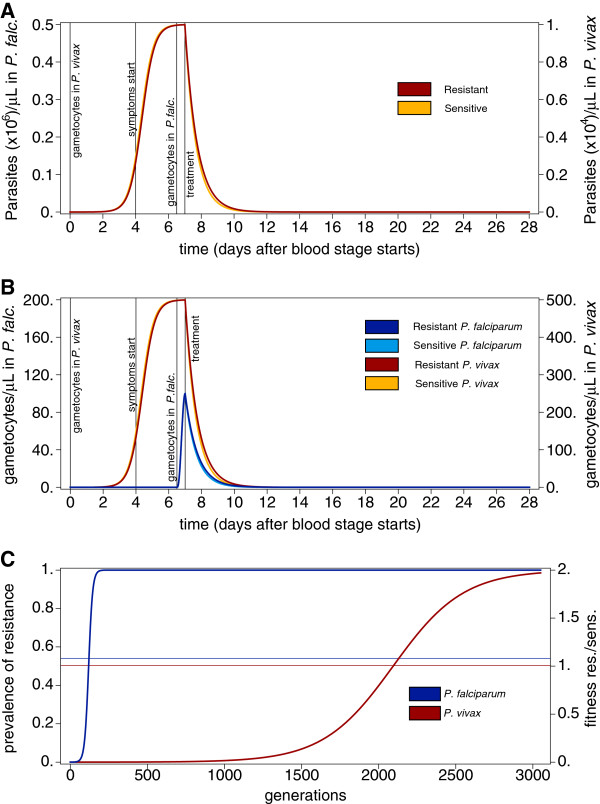
**Intra-host and population-level dynamics resulting from fitness components. A**) Population-average dynamics of asexual parasitaemia as a function of time after the erythrocytic cycle’s onset. They dynamics are shown separately for the resistant and sensitive parasites following equation (2). The y-axes on the left and right side correspond to the *P. falciparum* and *P. vivax* respectively. The dynamics are identical up to a scaling constant. On day seven drugs are administered to eliminate parasites. Resistant parasites are eliminated slightly less efficiently. **B**) Population-average gametocytaemia derived from the parasitaemia dynamics in A). Whereas, gametocytes are produced readily in *P. vivax*, they appear much later in *P. falciparum*. The dynamics are according to Equations (3) and (4). Vertical lines in **A**) and **B**) mark important events in a clinical episode. **C**) Spread of a resistant mutation - according to equation (1) - in *P. falciparum* and *P. vivax* is determined by the ratio of the areas under the curve of the gametocytaemia dynamics of resistant over sensitive parasites derived from **B**). Shown is the frequency of the resistant mutation as a function of time measured in generations corresponding to transmission cycles. The horizontal lines show the fitness ratio of resistant over sensitive parasites with corresponding right-hand side y-axis. Parameters are as described in Methods.

**Figure 2:**
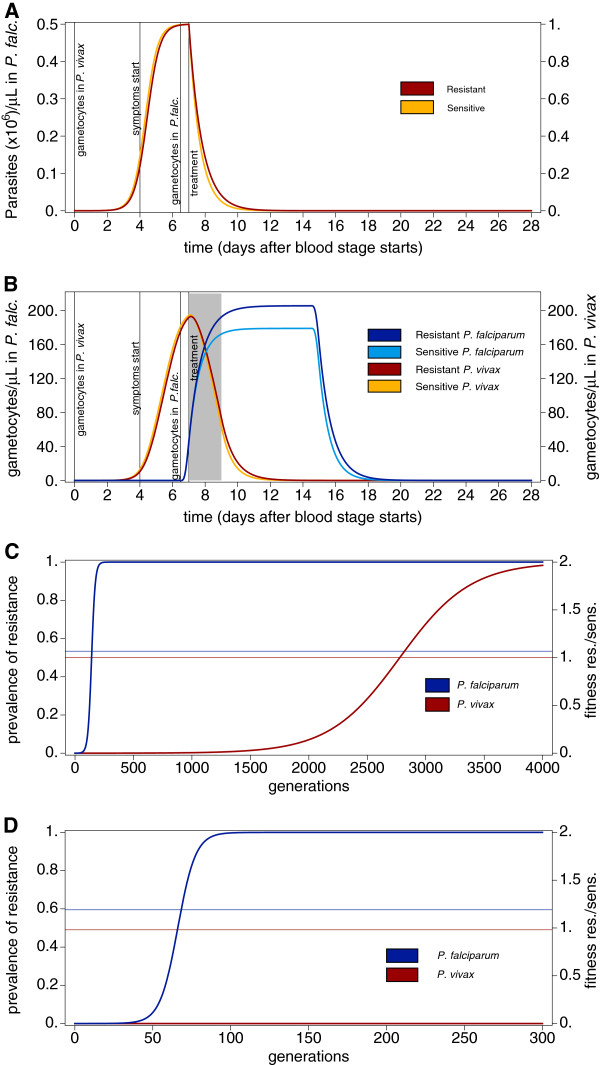
**Intra-host dynamics with refined gametocytaemia. A**) As Figure 1A with parameters described in Methods. **B**) As compared to Figure 1B, gametocytes are not just assumed to be proportional to merozoites but their longevity is incorporated – according to Equations (5) and (6) respectively. The grey shaded area indicates a period of isolation in which no transmission can occur, and is only relevant for figure D. **C**) as Figure 1C but fitness is derived from Figure 2B and (7). **D**) As in Figure C, but the two-day isolation (indicated in grey in Figure B) according to equation (8) is taken into account. Hence, the gametocytaemia dynamics in the grey shaded area are disregarded for the derivation of fitness.

For the present purposes, parasitaemia and gametocytaemia are considered as population averages. In a single malaria episode, parasitaemia increases in discrete cycles and will be under strong stochastic fluctuations. However, averaged over all malaria episodes in the population, parasitaemia will approximately follow a deterministic, continuous growth function (see Figures 1 and 2). The same holds for the gametocytaemia dynamics. In particular, not every malaria infection has gametocytaemia, but the population average will again follow a deterministic growth function.

In what follows ‘resistant’, ‘sensitive’, and ‘drug pressure’ can be substituted (with the necessary modifications) by ‘beneficial’, ‘deleterious’, and ‘selective force’, respectively, to obtain a more general interpretation. Resistant parasites might be associated with metabolic costs [17,18]. Hence, the population-average dynamics of resistant and sensitive parasites will be different, even in the absence of drug pressure. Notably, the emphasis of this population genetic model is on the ensemble of the various fitness components for each parasite species, not on the specifics of the parasitaemia and gametocytaemia dynamics. Overall, this population genetic model is used to compare *P. vivax* and *P. falciparum* in terms of how two specific fitness components, time of gametocyte production and their lifespan, affect the dynamics of an advantageous mutation expressed in the merozoite stage. Thus, the figures properly summarize the results. In order to contrast the importance that differences in gametocytaemia dynamics may have, a specific example on the effect of patient isolation may have on the spread of mutations associated with resistance in *P. vivax* and *P. falciparum* is discussed. The mathematical detail and the parameters chosen are described below. Readers shall feel free to skip the mathematical details as the figures guide the intuitive argument without mathematical formalism.

### Model

#### Evolutionary dynamics

Schneider and Kim [19,20] derived a model for the spread of resistant associated mutations tailored to the transmission cycle of *P. falciparum*. The model incorporated the possibility of co-infections, different numbers of sporozoites or sporozoite-strains infecting hosts, and heterogeneity among hosts (e.g., due to treated and untreated infections). Denoting the relative frequency of resistant parasites in generation *t* by *p(t)*, it was shown that the evolutionary dynamics follow the recursion equation

(1) $$p\left(t\right)=\frac{p\left(0\right)\lambda^{t}}{p\left(0\right)\lambda^{t}+\left(1-p\left(0\right)\right)\mu^{t}}=\frac{1}{1+\frac{1-p\left(0\right)}{p\left(0\right)}{\left(\frac{\lambda }{\mu }\right)}^{-t}}\text{,}$$

where time *t* is measured in discrete units of transmission cycles (i.e., one generations corresponds to one full transmission cycle - from sporozoite to sporozoite). Moreover, *λ* and *μ* are the fitnesses of resistant and sensitive parasites averaged over heterogeneous clusters of hosts with different responses to the infection, e.g., over treated and untreated hosts. The average fitnesses *λ* and *μ* are the likelihoods that, respectively, resistant or sensitive sporozoites causing an infection lead to gametocyte-offspring that are taken by a mosquito during its blood meal. Therefore, fitness is the results of various events that take place during the life cycle, the fitness components. Particularly, *λ* and *μ* depend on the average parasitaemia and gametocytaemia dynamics within infections. In the following, it is explained how the various fitness components assemble. From (1) it is clear that only the ratio $\frac{\lambda }{\mu }$ (the relation between the fitnesses of resistant and sensitive parasites) is considered in the evolutionary dynamics. If a mutation occurs that renders a parasite to be resistant – either in the human host or mosquito vector – initially only few sporozoites in one or a few mosquitoes will be resistant. Hence, if *N* denotes the number of sporozoites counted over all mosquitoes participating in a given transmission cycle, the initial frequency of resistant parasites *p*(0) is on the order of $\frac{1}{N}$. Notably, the formulation of the model incorporated co-infections by several mosquitoes. However, the dynamics and spread of resistant-associated mutations (at a single locus) are independent from the likelihood of co-infections (for more details see [19]). The situation would change with mutations interacting at two linked loci; however, such a more complex scenario is not considered at this point.

The transmission cycle for *P. vivax* is similar to that of *P. falciparum*, an important difference is that *P. vivax* infections involve liver-stage hypnozoites that can result in relapses. In a model that considers different merozoite variants [19,20], this can be approximated by assuming a higher likelihood of co-infections, since relapses will generate overlapping parasite generations. Ideally, the fitnesses of resistant and sensitive parasites in *P. vivax* need to be adjusted accordingly by considering the evolutionary dynamics of hypnozoites. This is especially important if hypnozoites are never treated in the population. However, such models involve different sets of assumptions that should be explored somewhere else.

### Parasitaemia dynamics

The most obvious process determining fitness is parasitaemia. Population-average blood-stage parasitaemia of, respectively, resistant and sensitive parasites in the absence of drugs is modelled according to the Holling Type III functions

$P_{S}\left(t\right)=\frac{P_{\max}}{1+\xi \rho_{S}^{-\tau }}$ and $P_{R}\left(\tau \right)=\frac{P_{\max}}{1+\xi \rho_{R}^{-\tau }}$

where *P*_max_ is the threshold parasitaemia, i.e., the maximum number of parasites that can be sustained within an infection. Here, *P*_S_ and *P*_R_ correspond to growth parameters of resistant and sensitive parasites, and *ξ* is a fitting parameter. The above dynamic imply exponential growth of sensitive and resistant parasites at low density after a slow initial establishment. However, once the parasitaemia approaches *P*_max,_ it will level off due to a carrying capacity. The Greek letter *τ* refers to the time scale of the duration of an average infection. Moreover, *τ* = 0 denotes the start of blood stage parasitaemia (erythrocytic cycle), and *τ* is scaled in days. The relation *ρ*_*S*_* > ρ*_*R*_ implies metabolic costs for resistance, i.e., a slower growth of resistant parasites [17,18].

After, the onset of drug treatment, parasitaemia will decrease exponentially. In particular, the dynamics are modelled by $P_{S}\left(\tau \right)=\frac{P_{\max}}{1+\xi \rho_{S}^{-\tau }}e^{-\delta_{s}\left(\tau -\tau_{0}\right)}$ and $P_{R}\left(\tau \right)=\frac{P_{\max}}{1+\xi \rho_{R}^{-\tau }}e^{-\delta_{R}\left(\tau -\tau_{0}\right)}$ for τ > τ_0_, where τ_0_ denotes the time that drugs become effective, and δ_S_, and δ_R_ denote the death rates of sensitive and resistant parasites, respectively. Naturally, δ_S_ > δ_R_ holds.

Moreover, *P*_max_ is replaced by *P*_max_^*falc*^ and *P*_max_^*vivax*^ to specifically model *P. falciparum* and *P. vivax*. Hence, apart from a scaling constant both parasite species are assumed to follow the same parasitaemia dynamics. In a more compact form these are given by

(2) $$P_{*}^{*}\left(\tau \right)=\frac{P_{*}^{*}}{1+\xi \rho_{*}^{-\tau }}f_{*}\left(\tau \right)$$

where $f_{*}\left(\tau \right)=e^{-\delta *\left(\tau -\tau_{0}\right)}$ for *τ > τ*_*0*_ and f_*_(*τ*) ≡ 1 for *τ > τ*_*0*_ (the asterisk in the subscript is a placeholder for *R* and *S*, and that in the superscript for *falc* and *vivax*).

The above parasitaemia dynamics are population averages including all treated and untreated infections.

### Gametocytaemia dynamics

While parasitaemia dynamics determine the performance of parasites within an average malaria episode, parasites’ fitnesses on a population scale are determined by their ability to transmit to other hosts. The fitness component determining transmissibility is summarized by the gametocytes’ dynamics. The population-average gametocytaemia, after its appearance, is assumed to be proportional to the population-average parasitaemia.

In *P. falciparum* episodes gametocytes appear at a later stage of the infection [1,14-16]. The average dynamics among *P. falciparum* infections are assumed to follow

(3) $$\begin{array}{l} \;G_{S}^{\mathrm{falc}}\left(\tau \right)=\varphi \varphi P_{S}^{\mathrm{falc}}\left(\tau \right)g^{\mathrm{falc}}\left(\tau \right)\;\mathrm{and}\;\\ \;G_{R}^{\mathrm{falc}}\left(\tau \right)=\varphi \varphi P_{R}^{\mathrm{falc}}\left(\tau \right) \end{array}$$

where *φ* is the percentage of blood-stage parasites developing into gametocytes. Moreover, *g*^*falc*^(*τ*) = 0 for *τ*_1_^*falc*^ >*τ* > 0 *g*^*falc*^(*τ*) = 64(*τ* − *τ*_1_^*falc*^)^3^*g*^*falc*^(*τ*) = 0 for *τ*_1_^*falc*^ >*τ* > 0, *g*^*falc*^(*τ*) = 64(*τ* − *τ*_1_^*falc*^)^3^(1 − *τ* + *τ*_1_^*falc*^)^3^ for $\tau_{1}^{\mathrm{falc}}+\frac{1}{2}>\tau >\tau_{1}^{\mathrm{falc}}$ and *g*^*falc*^(*τ*) = 1 for $\tau >\tau_{1}^{\mathrm{falc}}+\frac{1}{2}$. The function *g*^*falc*^*(τ)* ensures that gametocytaemia has a smooth onset over 12 hours and does not immediately jump from zero to *φP*_*max*_^*falc*^. This is mainly for illustrative purposes. The outcome of the model is independent of the particular choice of this function.

Gametocytaemia appears readily in *P. vivax* infections, i.e., at time *τ*_1_^*vivax*^ = 0 [1,14-16]. Hence, the gametocytaemia dynamics follow

(4) $$\begin{array}{l} \;G_{S}^{\mathrm{vivax}}\left(\tau \right)=vP_{S}^{\mathrm{vivax}}\left(\tau \right)f_{S}\left(\tau \right)g^{\mathrm{vivax}}\left(\tau \right)\;\mathrm{and}\;\\ \;G_{R}^{\mathrm{vivax}}\left(\tau \right)=vP_{R}^{\mathrm{vivax}}\left(\tau \right)f_{R}\left(\tau \right)g^{\mathrm{vivax}}\left(\tau \right) \end{array}$$

where *v* is the percentage of blood-stage parasites developing into gametocytes in *P. vivax* and *g*^*vivax*^*(τ)* is defined as *g*^*falc*^*(τ)* with *τ*_1_^*falc*^ replaced by *τ*_1_^*vivax*^.

Note, that the functions g*(*τ*) account for the different onsets of gametocytogenesis in *P. falciparum* and *P. vivax*. Namely, gametocytogenesis starts with the erythrocytic cycle in *P. vivax*, (about a week before drug treatment starts). However, in *P. falciparum* gametocytogenesis starts shortly before drug treatment starts [1,14,16].

### Life-span of gametocytes

Alternatively to the above, more realistic gametocytaemia dynamics can be assumed by including an additional fitness component. Namely, gametocytes are non-reproducing; they derive from merozoites and die after a while if they are not transmitted by a mosquito vector.

Hence, the number of gametocytes at time *τ* in, respectively, *P. falciparum* and *P. vivax* are

(5) $$G_{*}^{\mathrm{falc}}\left(\tau \right)=\int_{\mathrm{Max}\left[0,\tau -\beta_{\mathrm{falc}}\right]}^{\tau }\varphi \varphi P_{*}^{\mathrm{falc}}\left(z\right)f_{S}\left(z\right)g^{\mathrm{falc}}\left(z\right)\mathrm{dz}$$

and

(6) $$G_{*}^{\mathrm{vivax}}\left(\tau \right)=\int_{\mathrm{Max}\left[0,\tau -\beta_{\mathrm{vivax}}\right]}^{\tau }\nu P_{*}^{\mathrm{vivax}}\left(z\right)f_{*}\left(z\right)g^{\mathrm{vivax}}\left(z\right)\mathrm{dz}$$

where *β*_*vivax*_ and *β*_*falc*_ are the life spans of gametocytes in *P. vivax* and *P. falciparum*, respectively.

### Fitness

The various fitness components can be combined to determine fitness. The fitness of a parasite is the probability that a gametocyte offspring is transmitted to a different host, and hence can further participate into the transmission cycle. Any gametocyte can be picked up by a mosquito whenever gametocytaemia exceeds a threshold *G*_*crit*_^*vivax*^, or *G*_*crit*_^*falc*^ for *P. vivax* or *P. falciparum*, respectively. Hence, the fitnesses of parasites are proportional to the respective mean value of the average gametocytaemia, which is taken over the time window when gametocytaemia exceeds its threshold. Hence fitness is proportional to $\int_{a_{*}^{*}}^{b_{*}^{*}}G_{*}^{*}\left(\tau \right)\mathrm{d\tau}$.

Here, (*a*_*R*_^*vivax*^, *b*_*R*_^*vivax*^), (*a*_*S*_^*vivax*^, *b*_*S*_^*vivax*^), (*a*_*R*_^*falc*^, *b*_*R*_^*falc*^), (*a*_*S*_^*falc*^, *b*_*S*_^*falc*^), denote the time intervals in which resistant and sensitive gametocytes exceed the threshold gametocytaemia to be transmittable in *P. vivax*, and *P. falciparum*, respectively. This implies a density-dependence in fitness.

Thus, the fitness advantage of resistant over sensitive parasites in *P. vivax* and *P. falciparum* are

(7) $$\frac{\lambda }{\mu }=\frac{\int_{a_{R}^{*}}^{b_{R}^{*}}G_{R}^{*}\left(\tau \right)\mathrm{d\tau}}{\int_{a_{S}^{*}}^{b_{S}^{*}}G_{S}^{*}\left(\tau \right)\mathrm{d\tau}}\text{.}$$

Note, that in Schneider and Kim [19] λ and μ are averaged over treated and untreated hosts (or heterogeneous clusters of hosts). Here this is already accounted for by regarding the population-average parasitaemia and gametocytaemia dynamics.

### Isolation of patients

As a standard healthcare procedure, malaria patients might be isolated by using bed nets. During isolation, malaria cannot be transmitted, which results in an additional fitness component. Hence, this needs to be accounted for when deriving fitnesses of resistant and sensitive parasites on a population level. In particular, if the patient isolation is sustained For τ_*Q*_ days, the fitnesses are proportional to $\int_{a_{*}^{*}}^{b_{*}^{*}}G_{*}^{*}\left(\tau \right)I_{\left[\tau_{0},\tau_{0}+\tau_{Q}\right]}\left(\tau \right)\mathrm{d\tau}\text{,}$ and the fitness ratios become

(8) $$\frac{\lambda }{\mu }=\frac{\int_{a_{R}^{*}}^{b_{R}^{*}}G_{R}^{*}\left(\tau \right)I_{\left[\tau_{0},\tau_{0}+\tau_{Q}\right]}\left(\tau \right)\mathrm{d\tau}}{\int_{a_{S}^{*}}^{b_{S}^{*}}G_{S}^{*}\left(\tau \right)I_{\left[\tau_{0},\tau_{0}+\tau_{Q}\right]}\left(\tau \right)\mathrm{d\tau}}\text{.}$$

Here, $I_{\left[\tau_{0},\tau_{0}+\tau_{Q}\right]}\left(\tau \right)=1$ for *τ*_*0*_*< τ < τ*_*0*_*+ τ*_*Q*_ and 0 otherwise. In other words, whereas gametocytaemia during the patient isolation period is assumed irrelevant in terms of transmission, isolation of patients changes the relative fatnesses of resistant versus sensitive parasites. Of note, the changes in fitness are qualitatively and quantitatively different in *P. falciparum* and *P. vivax*, since dynamics of gametocytaemia are different between these species.

### Parameter choice

To lay out the formal arguments, parameters are chosen that are intuitive and lead to within-host and evolutionary dynamics that biologically meaningful.

First, note that the ratio of resistant and sensitive fatnesses or $\frac{\lambda }{\mu }$ is independent of the choice of *P*_max_^*vivax*^, *P*_max_^*falc*^, *v*, and* φ*, *if G*_*crit*_^*vivax*^ and* G*_*crit*_^*falc*^ are properly scaled. Hence, the merit of keeping these parameters is to provide examples with realistic values for gametocytaemia and parasitaemia when *P. falciparum* and *P. vivax* are contrasted. Moreover, note that the parameters were chosen to achieve illustrative and representative examples. Hence, their concrete values are of secondary importance.

The following parameters determining parasitaemia dynamics are assumed: *P*_max_^*vivax*^ = 10^4^/*μL*, *P*_max_^*falc*^ = 5 * 10^5^/*μL*[21], and *ξ*=10^5^ For the basic model determined by Equations (1) – (4) the parameters $\rho_{S}=\frac{100}{7},\;\rho_{R}=\frac{100}{7.25},\;\delta_{S}=1.5\;\mathrm{and}\;\delta_{R}=1.35$ are used, which implies slight metabolic cost for resistance. For the model accounting for gametocytes’ life-span, $\rho_{S}=\frac{100}{7},\;\rho_{R}=\frac{100}{7.5},\;\delta_{S}=1.5\;\mathrm{and}\;\delta_{R}=1.25$ are used. These choices guarantee that the maximum parasitaemia will be nearly reached after five to seven days after the onset of the erythrocytic cycle. Further, treatment is assumed to start at *τ*_*0*_=7.

Regarding gametocytaemia specific parameters, the onset of gametogenesis in *P. vivax* is immediately after the start of the erythrocytic cycle, i.e., *τ*_1_^*vivax*^ = 0, whereas it is 6.5 days later in *P. falciparum*, i.e., *τ*_1_^*falc*^ = 6.5. The gametocytaemia threshold for transmission is assumed to be *G*_*crit*_^*vivax*^ = *G*_*crit*_^*falc*^ = 10/*μL*.

In the basic model *v* = 0.05, and φ = 0.0004 were used. In the model accounting for gametocytes’ life-span *v* = 0.01, and φ = 0.0004 were used. Moreover, longevity of gametocytes is set to 8 days for *P. falciparum* (*β*_*falc*_ = 8, [22]) and 2 days for *P. vivax* (*β*_*vivax*_ = 2; [23]).

When isolation of treated patients is incorporated, 2 days of isolation are assumed, i.e., *τ*_*Q*_ = 2 in equation (8).

## Results

### Dynamics for the simple model

The argument that is laid out here assumes quantitatively identical parasitaemia dynamics for *P. falciparum* and *P. vivax* episodes. Even further, the dynamics are identical up to a scaling constant to ensure meaningful merozoite counts for both species. The merit of this approach is to keep some fitness components constant allowing contrasting the impact of the remaining components. Figure 1A shows the population-average parasitaemia dynamics of resistant and sensitive parasites in *P. falciparum* and *P. vivax*. Note, that resistant parasites are just slightly less efficiently removed than sensitive parasites. (In fact such differences might not even manifest in clinical outcomes).

Gametocytes are produced at earlier stages in *P. vivax* infections when exposure to antimalarial drugs is unlikely. Hence, during an infection, the time window in which sensitive parasites are selectively advantageous and produce gametocytes at the same time is prolonged in *P. vivax*. This is depicted in Figure 1B. Therefore, over the course of the infections, disproportionally more sensitive gametocytes contribute to *P. vivax* transmission when compared to *P. falciparum*. Consequently, this translates into smaller fitness differences between resistant and sensitive parasites in *P. vivax*, since fitness is determined as the overall prevalence of gametocytes during the population average course of infections. The dynamics for the spread of resistance prevalence is depicted in Figure 1C. While under these circumstances resistance spreads within 200 generations in *P. falciparum* it takes 3,000 generations in *P. vivax*. This corresponds to 20–40 years in *P. falciparum* and 300–600 years for *P. vivax*, assuming 5 to 10 malaria generations per year. This notorious difference is observed by simply considering that gametocytes in *P. vivax* are produced earlier than in *P. falciparum* during the course of an infection.

### Incorporation the life span of gametocytes

The argument as laid out above, still applies if the differences in the life-span of gametocytes is incorporated (Equations 5 and 6), as shown in Figure 2. From similar parasitaemia dynamics (Figure 2A), the gametocytaemia dynamics are derived (Figure 2B). Again, the prevalence of resistant compared with sensitive gametocytes is higher in *P. falciparum* episodes, resulting in stronger selection for drug-resistance, and faster spread of resistance prevalence in *P. falciparum* than in *P. vivax* (cf. Figure 2C).

### Isolation of patients

Surprisingly, the strength of selection for drug resistance and hence its spread, will increase by short isolation measures (Equations 7 and 8) in clinical episodes of *P. falciparum*, whereas it can be an effective mechanism to prevent the spread of drug-resistance in *P. vivax*. Figure 2D shows the spread of resistance derived from the gametocytaemia dynamics for Figure 2B when two-day isolation is assumed in clinical cases (see Methods). Hence, the fitnesses are calculated as for Figure 2C, except that the dynamics two days after the start of treatment do not contribute to determine fitness - grey shaded area in Figure 2B. Isolation prevents the spread of drug resistance in *P. vivax*, since gametocytes have a short lifespan and they will likely die after the onset of drug-treatment.

In *P. falciparum* episodes the situation differs because gametocytes are longer lasting. Hence, during isolation, proportionally the transmission of sensitive gametocytes is more severely reduced than that of resistant gametocytes. Shortly after the onset of drug treatment, more sensitive than resistant gametocytes could be transmitted, which however, is precluded precisely by the isolation. By comparing Figure 2C with Figure 2D it becomes clear that the spread of drug resistance is facilitated by short-term isolation measures. Namely, it spreads within 100 generations instead of 200 generations, corresponding to 10–20 years compared with 20–40 years when assuming 5–10 generations (transmissions cycles) per year. Notably, these results are very sensitive on the duration of isolation. In principle, as in *P.* vivax, resistance evolution can be prevented in *P. falciparum* if the isolation period exceeds the longevity of gametocytes, i.e., if isolation would be sustained for several weeks. However, such possibility has no basis in reality.

## Discussion

The impact of interacting parasitaemia and gametocytaemia dynamics on drug-resistance evolution was formally studied. Namely, the contribution of various fitness components to the overall fitness of sensitive and resistant parasites was explored. Their basic components are, (i) growth and decay rates under drug pressure on merozoites, (ii) the starting point of gametocytogenesis, (iii) the duration of gametocytes in the blood stream, (iv) threshold gametocytaemia for transmissibility, and, eventually, (v) isolation periods of clinical malaria episodes. The fitnesses of resistant and sensitive dynamics are ultimately calculated from the area under the curve of the gametocytaemia dynamics over the time window during which gametocytes can be transmitted. A similar reasoning to measure fitness was introduced earlier [24].

Importantly, the assumptions on the parasitaemia and gametocytaemia dynamics made here are by no means crucial, since they affect the results only quantitatively but not qualitatively. Indeed, in the illustrative examples presented here, reasonable values for the parameters were used from the literature, which might not exactly agree with every other study. However, for the arguments laid out here, only the qualitative picture that gametocytes occur earlier in *P. vivax* but persist longer in *P. falciparum* matters. Indeed this was confirmed in many studies (despite ambiguous values among studies) [1,14-16]. Overall, what matters is that late gametocytogenesis during the infection and longer lifespan of gametocytes will promote the spread of drug resistance. Thus, when contrasting these two malarial parasites, resistance will spread easier in *P. falciparum* than in *P. vivax*. It shall be pointed out that the role of hypnozoites was roughly approximated in terms of a higher prevalence of co-infections in *P. vivax* than in *P. falciparum*. The role of hypnozoites, especially if they are not properly treated, should be explored elsewhere.

For simplicity, the methods and results were tailored towards resistance against drugs that target solely the merozoite stage; however, an almost identical reasoning implies that any traces of selection occurring acting on blood stage merozoites will be more visible for *P. falciparum* (gametocytogenesis; longevity of gametocytes).

Based on the results presented here, the use of drugs that can clear gametocytes, e.g., primaquine (PQ), could prevent the spread of drug-resistant solely on the basis of its effect on critical fitness components. Whereas one could make a solid argument about its benefits based on its blocking transmission effect, the proposed decomposition of fitness into basic components allows for understanding the potential benefits of using this drug in terms of how selection acts on mutations associated with resistance at the population level. By clearing gametocytes from parasites with mutations that confer resistance against the primary drug, their likelihood of transmitting will be reduced reducing their fitness advantage compared to sensitive parasites. Hence, PQ use can potentially impede or delay the onset and spread of resistant mutations by having an effect on the evolutionary dynamics of such mutations.

Transmission is the ultimate measure of the parasites’ fitness so, at this level, epidemiologic and evolutionary arguments are expected to converge to the same conclusions as shown in this investigation. However, the results here highlight the importance of gametocyte dynamics with a different perspective. Investigating gametocyte dynamics is not only important in the usual context of understanding and blocking transmission in an epidemiologic sense, but it is also critical in the context of sustaining successful malaria control strategies. Indeed, gametocyte dynamics determines how those control tools (e.g. antimalarial drugs) act as selective forces on the parasite population compromising their long-term efficacy. Namely, they affect the spread of mutations that are selected by interventions, in this case, drug resistance. Therefore, more detailed studies on the gametocytaemia dynamics are needed. Unfortunately, most studies reported only on the prevalence of gametocytes in clinical episodes on certain census points after the beginning of treatment. However, a description of population-average gametocytaemia as employed here would be highly desirable. Since such studies do not exist, we were unable to tailor our model towards more realism. Furthermore, existing studies [15] are often based on data from neurosyphilis patients, and hence may not be representative for an average endemic area. However, as argued above, the qualitative picture will remain unchanged as it is independent of the particular choice of gametocytaemia parameters.

Whereas this model focused on comparing two parasites with clear differences in their gametocytaemia dynamics, its findings regarding the interplay between the efficacy of selection acting on mutations associated with drug resistance and gametocytaemia should be considered in a broader context. E.g. it is important to consider that the prevalence and density of gametocytaemia are affected by particular drug treatments. For instance, Robert *et al.*[25] showed that gametocytaemia prevalence and density is substantially higher in SP treated patients than in CQ treated patients. Possibly, SP even triggers gametocytogenesis or CQ prevents gametocytogenesis. On the other hand, artesunate seems to reduce gametocytaemia prevalence substantially in the course of ACT [26-30]. On the contrary, it seems that amodiaquine (AQ) - administered as AQ or AQ+SP - leads to high gametocytaemia prevalence compared with other treatments [26,31-34]. Thus, it seems that there is evidence indicating that some combination drug therapies could be prone to spread resistant mutations by affecting gametocytaemia under circumstances that need to be properly explored. Detailed studies on the population average gametocytaemia under different drug treatments, and whenever sensitive and resistant/tolerant parasites are present, would provide desirable information to refine our understanding of resistance evolution.

## Conclusions

By contrasting *P. vivax* and *P. falciparum*, this investigation illustrates how differences in fitness components, time of gametocyte production and their lifespan, have a critical effect on the efficacy of selection acting on mutations that express their phenotypic effect at the merozoite stage in these two parasite species. Overall, selection at the merozoite stage is more effective in *P. falciparum* than in *P.* vivax. These findings could explain, at least in part, the observed differences in terms of the spreading of drug resistance between these two major malarial parasites.

The proposed model allowed exploring the differential effect of isolating patients on these two species in terms of the spread of mutations associated with drug resistance. Even further, if transmission can be affected by other suitable measures (other than isolation), then the rise in frequency of advantageous mutations associated with drug resistance at the merozoite stage will be delayed or reduced drastically depending on the relative role of the different fitness components. Like in previous epidemiologic or population-biology models, this approach suggests that by employing drugs that successfully reduce gametocytes, the onset of drug resistance could be delayed (e.g. using primaquine to contain the spread of mutations associated with drug resistance or tolerance in *P. falciparum*). Noteworthy, this argument is more refined than the usual transmission-blocking argument, namely it consists of two parts: (i) by clearing parasites while they are advantageous against the primary drug, the fitness of resistant mutations is effectively reduced; (ii) reduced gametocytaemia reduces the likelihood of transmitting malaria, hampering the onset and spread of resistance.

Whereas this investigation focused in compared very different gametocyte dynamics (*P. vivax* against *P. falciparum*), it emphasizes the importance of gametocytaemia in terms of the strength of selection acting on the parasite. Overall, there is a need of monitoring gametocytaemia more carefully over long periods at a population level (note that gametocytes might be carried up to 55 days [25]). Moreover, more studies on the mechanisms that may trigger gametocytogenesis (e.g. specific drug combinations) and the importance of submicroscopic gametocytaemia seem highly desirable.

## Competing interest

The authors declare that they have no competing interests.

## Authors' contributions

KS and AE designed the investigation. KS adapted the mathematical model and performed the simulations. KS and AE discussed the analyses and drafted the manuscript. Both authors read and approved the final manuscript.
